# Analyzing and Validating a Structure for Measuring the Nurse Practice Environment

**DOI:** 10.3390/ijerph20075266

**Published:** 2023-03-27

**Authors:** John Rodwell, Thomas Hendry, Dianne Johnson

**Affiliations:** 1 Department of Management and Marketing, Swinburne University of Technology, Melbourne, VIC 3122, Australia; 2 Griffith Business School, Griffith University, Brisbane, QLD 4111, Australia

**Keywords:** practice environment, nurses, Magnet, predictive utility, job design

## Abstract

Nurse shortages pose a challenge in many countries and retaining existing nursing staff is crucial to addressing these shortages. To inform possible interventions aimed at retaining nurses, managers need a comprehensive understanding of the nature of the nurse practice environment. The scales from two of the main instruments used to assess nurses’ practice environments are tested. A survey of an online panel obtained responses from 459 Australian nurses. Analyses determined a combination of items with good construct validity and improved predictive utility for outcomes of interest for individual nurses. By essentially combining the best items from each instrument, a more comprehensive representation of the nurse work environment is obtained with improved predictive utility. The resulting combined set of scales is recommended for analyses of the nurse working environment and uses a combined set of scales from each of the two source instruments, namely: nurse participation in hospital affairs, recognition, nursing process, peer work standards, nursing competence, orientation, managers, resources, nurse–physician collaboration, and positive scheduling climate. Future research can then build on that strong set of items with a validated structure and predictive utility to inform management and interventions.

## 1. Introduction

Nurse shortages pose a challenge to many developed countries today, where nurses are crucial to delivering high-quality health assessments, care, and treatments to citizens [1,2]. Moreover, global demand for nurses is predicted to rise over the next decade [1]. Recent estimates suggest that around 9 million new nurses and midwives will be required by 2030 [2]. In this context, retaining existing nursing staff (and encouraging new entrants) is crucial to addressing current and future supply shortages of nurses worldwide. A supportive practice environment for nurses (PEN) has long been recognized as a key factor in retaining nurses [3]. A positive PEN is conceptualized as enhancing nurse job satisfaction and patient care quality by empowering nurses with higher levels of autonomy [4]. The nursing practice environment allows nurses to achieve a high level of clinical practice, providing greater efficacy in mobilizing available resources, while working in an interdisciplinary team [4]. The nursing practice environment allows nurses to provide more effective preventative treatments and monitoring for patients [5]. Such higher quality clinical care is achieved in a practice environment that empowers nurses with increased autonomy, accountability, and decision making [5].

A positive PEN is seen to be a function of implicit and explicit organizational factors such as leadership, collaborative processes, and professional practices [4,5,6,7,8], and it has widespread benefits such as increased levels of nurse empowerment [8], higher work satisfaction [9,10], reduced intention to leave [11], higher job enjoyment [12], and higher quality patient care [13]. Key outcomes such as job satisfaction in turn affect nurse retirement behavior [14].

Given the multidimensional nature of PEN and its importance to several positive outcomes, the reliability and validity of its measurement is vital. The PEN is thus measured using specifically designed instruments containing subscales of relevant domains. These multidimensional instruments provide a mechanism to compare work environments, predict outcomes (related to patients, staff, and organizations), and inform policy interventions, ultimately assisting nurses to practice more effectively and provide higher quality patient care [15].

### Literature Basis of the Main Instruments Assessing Nurses’ Practice Environment

Several instruments have been designed to measure PEN that vary based on their purpose and specific item content [4]. Of these, the Revised Nursing Work Index (NWI-R) [16] and the Practice Environment Scale of the Nursing Work Index (PES-NWI) [17] are the most widely used instruments. The NWI-R is an expanded version of the original Nursing Work Index (NWI) developed during the 1980s, which includes 65 questions primarily focused on job satisfaction and care quality in hospitals [18]. The revised version (NWI-R) further captures organizational characteristics such as management style, leadership, and professional development by omitting certain questions from the NWI, while adding new subscales to address these organizational characteristics, resulting in a revised 57-item instrument [16].

An initial set of items was identified through a search of the Cumulative Index to Nursing & Allied Health Literature database, for articles published in the period of 1996–2005 [4]. Instruments from these studies were classified based on the key development features of each instrument (e.g., discipline, purpose, source) and subsequently assessed based on theoretical relevance, ease of use, and body of evidence [4]. The broader history of the development of the practice environment scale by Lake [17] and colleagues from the Nursing Work Index data from Kramer and Aiken’s research is detailed in [5], where the broader pool of items was analyzed with an exploratory factor analysis and then in both Magnet and non-Magnet hospitals.

Organizational characteristics are included as an expansion of the original NWI because they are associated with nurse job satisfaction [9], reduced intention to leave [11], and subsequent job enjoyment and patient outcomes [12,19]. The NWI-R can be modified to a country-specific context, such as by Anunciada et al., 2022, who identify the NWI-R as a reliable and valid instrument for characterizing the nursing practice environment in Portugal [20].

The PES-NWI was developed based on the factor analysis of a sample of nurses working in Magnet-certified hospitals from the original NWI data [17]. The PES-NWI consists of five subscales: nurse participation in hospital affairs, nursing foundations for quality of care, nurse manager ability, leadership and support of nurses, staffing and resource adequacy, and collegial nurse–physician relations [4,17]. The usefulness of the PES-NWI was considered in a subsequent study by Lake (2007) [4] assessing the utility of seven multidimensional instruments (including the NWI-R), based on theoretical relevance, ease of use, and body of evidence, and identified the PES-NWI as the most useful. Furthermore, reviewing 37 studies, Warshawsky and Havens (2011) report a strong association between the PES-NWI and a range of outcomes at the nurse, patient, and organizational levels [21]. For example, nurses’ perception of patient safety is associated with several subscales in the PES-NWI [22], while several subscales are also associated with resilience in new nurses [23]. The frequency of use of the PES-NWI in subsequent research has remained high [15,24] and is robust to nursing settings in countries outside the U.S [25,26,27,28]. The PES-NWI can also be modified to capture heterogeneity across countries: for example, the PES-AUS developed by Middleton et al. (2008) for the Australian nursing context [29].

Although the NWI-R and the PES-NWI are widely used, extensions to these instruments can increase their coverage of the domain of the nurse working environment as well as improve reliability and validity. One such extension of the NWI-R proposed by Choi et al. (2004) considered the measurement of the working environment as perceived by nurses, resulting in a seven-factor instrument, the Perceived Nursing Work Environment (PNWE) [5]. Given the psychometric properties of the PNWE, the instrument may also complement the PES-NWI, which others have suggested could benefit from additional psychometric testing, updating, and development [15].

The insights garnered from these instruments have guided the development of the Magnet Recognition Program—a certification framework recognizing working environments that demonstrate an excellent standard in nursing practices and strategies [30]. Certification is awarded based on five components referred to as the five forces of magnetism: transformational leadership, structural empowerment, exemplary professional practice, new knowledge, innovation and improvements, and empirical quality results [31]. Magnet certification has an emphasis on attracting (and retaining) nurses to the organization and has been associated with the organization upholding excellent standards in quality care, continuing professional development of staff, fair hierarchal employment structures, effective staff deployment, and high job satisfaction [32]. Magnet certification has been found to be associated with several outcomes such as reduced nurse burnout, reduced intention to leave, and lower staff turnover [4,33,34], while also increasing nurse engagement in hospital affairs [4,35]. These outcomes at the nurse level are also associated with the quality of patient care. For example, Schiak et al. (2021) found that Magnet-certified hospitals mediate the effect of nurse burnout on patient mortality and failure to rescue [13].

Earlier authors have noted that policy making to address nurse shortages and promote retention should avoid relying on overly simplistic thinking [1]. To that end, instruments such as the NWI-R, PES-NWI, and PNWE are examples of sophisticated scales that measure various elements of PEN, thereby attempting to capture the many facets of the nurse working environment. However, revising and improving these scales is worthwhile insofar as it creates a better understanding of PEN. Specifically, combining subscales from multiple instruments may uncover novel insights about PEN and assist subsequent policy makers to increase retention, thereby helping to address nurse shortages.

To assess the predictive utility of the scales derived to more comprehensively and efficiently cover the domain of PEN, a variety of outcome measures are used, including several with strong but differing relationships with retaining nurses and measures of performance behaviors. The analyses against the outcome variables mainly assess predictive utility, but also confirm other psychometrics of the scales such as further evidence of discriminant and convergent validity (where the scales created from the factors correlate with the scales they should correlate with and do not correlate with the scales that they should not correlate with). The outcomes investigated in this study range from individual-oriented indicators of labor turnover (intent to quit and job satisfaction), to organization-driven indicators of labor turnover (organizational commitment), to indicators of performance behaviors, as well as occupational indicators of commitment to their occupation (occupational commitment).

Job satisfaction and intent to quit have often been used in assessing the characteristics of the nurse practice environment that precede nurses leaving the organization (e.g., [16,18]). However, some studies have found no links between previous incarnations of PEN instruments, such as collegial nurse–physician relations, nurse manager ability, leadership and support, and nurses’ turnover intentions (e.g., [36]).

A less commonly studied outcome for research on the PEN is affective organizational commitment, which is often found to be a good precedent of employees quitting or their willingness to stay with the organization, across a variety of industries [37]. Affective organizational commitment is sometimes considered as being more discretionary by health services employees, yet it can be especially relevant to health services organizations where the level of service is difficult to maintain without a strong emotional commitment to the organization and its goals [38].

Perhaps the more novel outcomes to be predicted by the PEN scales assessed in this study are those associated with performance behaviors, whether those behaviors are extra-role, that is, outside the nurse’s job description, or whether those behaviors are in-role. The extra-role behaviors are often referred to as organizational citizenship behavior (OCB) and are important for the effective functioning of organizations [39]. OCBs may be directed toward individuals (OCBI) or at the organization (OCBO) [40]. In nursing, OCBs are a particularly important outcome because nurses have the most frequent interactions with patients, and their positive behaviors can strongly influence the quality of healthcare services [41].

The first stage of this study investigates whether complementing the items of the PES-NWI with the extra items from the PNWE increases the coverage of the domain of the nurse practice environment. That assessment of the comprehensive representation of PEN entails developing a measurement model across the array of items, with a particular emphasis on the construct validity of the resulting factors, as well as the discriminant and convergent validity of the factors. The second stage of the study entails assessing the reliability of the scales, the pattern of convergent and discriminant relationships across the scales, but particularly assessing the predictive utility of the scales in terms of predicting key variables of interest such as intent to quit, affective organizational commitment, affective occupational commitment, job satisfaction, and performance behaviors such as OCBI, OCBO, and in-role performance behavior (IRB).

## 2. Method

### 2.1. Sample Recruitment

Nurses were recruited using PureProfile, an online web panel consisting of 550,000 Australians, almost three percent of the entire Australian adult population. All members of the online panel who were over 18 and having previously indicated that their occupation was as a nurse were invited to participate in the survey via email, whereby participation was indicative of consent. Further, filter questions were used before a respondent could access the survey, where the respondents had to confirm that they were over 18 and had been working as a paid nurse in Australia during the prior month. Following completion of the survey, PureProfile paid respondents AUD 5.

Online panels provide several advantages to researchers, including targeted sampling of low-incidence groups, access to a demographically representative pool of respondents, and previously collected background data on participants [42,43]. For these reasons, online panels are increasingly being used in research studies (e.g., [44]).

### 2.2. Sample Characteristics

The sample comprised 459 Australian nurses and their characteristics are detailed in Table 1. The nature of the respondents was compared to the national profile of nurses in Australia [45]. The sample was different from the population of nurses in terms of their age (χ^2^(4) = 52.47, *p* < 0.001), in that it had a younger profile with notably more 25–34 year-olds and relatively less nurses 55 years old and over. The sample was different from the population of nurses in terms of gender (χ^2^(1) = 40.63, *p* < 0.001), in that it had a higher proportion of males than the population, although the number of males in the sample was still low and this difference may also be a reflection of males comprising more of the younger nurses in the nurse population (i.e., that gender difference could be because of the younger age profile). The other distinction was that the sample had a lower proportion of RNs (χ^2^(1) = 67.12, *p* < 0.001; 66.4% cf 81.3%) than the proportion in the nursing population, which may also be a reflection of the younger age profile. The authors are not aware of any studies showing that the factor structures of PEN scales vary by age (or gender) and note these differences for future analyses and as a possible basis of limitations. The study was approved by the Ethics Committee at Deakin University and then of the Australian Catholic University EC-206V, including extensions. All respondents participated voluntarily, where the responses were anonymous and unidentifiable by the researchers.

### 2.3. Measures

This study first investigates whether the items of the PES-NWI with the extra items from the PNWE increase the coverage of the domain of the nurse practice environment. The second stage of the study entails assessing the reliability of the scales and the pattern of convergent and discriminant relationships across the scales, particularly assessing the predictive utility of the scales in terms of predicting key variables of interest such as intent to quit; affective organizational commitment; affective occupational commitment; job satisfaction; and performance behaviors such as OCBI, OCBO, and IRB.

Perceived Nursing Work Environment. This 42-item scale contains the seven subscales of nursing management, staff and resource adequacy, nursing process, nurse–physician collaboration, nursing competence, and positive scheduling climate [5]. A four-point rating was used for each item forming the Likert subscales (1 strongly disagree, 4 strongly agree).

Perceived Environment Scale—Nursing Work Index. This is a 31-item scale containing five subscales, namely, nurse participation in hospital affairs; nursing foundations for quality of care; nurse manager ability, leadership and of support nurses; staffing and resource adequacy; and finally, collegial nurse–physician relations [17]. A four-point rating was used for each item across these Likert subscales (1 strongly disagree, 4 strongly agree).

For the second stage of the analyses, a variety of outcome scales were included in order to conduct later validity checks, especially for the predictive utility of the subscales determined by the measurement model analyses of the nurse working environment instruments. The outcomes scales range from individual-oriented indicators of labor turnover (intent to quit and job satisfaction), to organizational indicators of labor turnover (organizational commitment), to indicators of performance behaviors and occupational indicators of commitment to their occupation (occupational commitment).

Intent to quit. Two items were taken from Landau and Hammer (1986) [46], one item was adapted from Wayne et al. (1997) [47], and four items were taken from Chatman (1991) [48] to measure intent to quit. The items used from [46] were: ‘I am actively looking for a job outside my organization’ and ‘I am seriously thinking about quitting my job’. The original item from [47] was I think I will be working at [company name] five years from now, which was made applicable across organizations by being changed to “I expect to be working at this organisation two years from now”. The four items from [48] were: “[W]ould you prefer another more ideal job than the one you now work in”, “I have thought seriously about changing organizations since beginning to work here”, “I intend to remain with this organization”, and “[I]f I had my way, I will be working for this organization three years from now”. All items were scored on a five-point Likert rating (1 strongly agree, 5 strongly disagree).

Affective organizational commitment. This is an eight-item scale [49] with responses on a five-point Likert rating (1 strongly disagree, 5 strongly agree).

Affective occupational commitment. This is a six-item scale [50] using a five-point Likert scale (1 strongly disagree, 5 strongly agree).

Job satisfaction. This is a six-item scale as used by Agho, Prince, and Mueller (1992) [51], with response options on a five-point Likert rating (1 strongly disagree, 5 strongly agree).

Performance Oriented Behaviors. Three scales of seven items each were used to represent a range of performance-oriented behaviors: those behaviors representing performative activities beyond their job’s formal roles—OCBI and OCBO, as well as an assessment of behaviors within their role—IRB [40]. All of the 21 performance behavior items are rated on a seven-point rating (1 strongly disagree, 7 strongly agree) to form Likert scales.

### 2.4. Data Analytic Approach

The instrumental reliability (inter-item reliability and internal consistency) and validity of the instruments were assessed following the approach of [52], which is very similar to the later COSMIN definitions [53], along with the appropriate statistical checks (detailed in [54,55,56]). The broad assessment of the validity of the instruments reflects a variety of components (see [52,53] for further detail).

A component of the assessment of content validity is the assessment of face validity [53]. Face validity is the subjective process where judges assess that the items measure what they claim to measure [52]. Throughout the analyses detailed below, the statistical considerations are combined with an ongoing judgement of face validity with regard to whether a particular item reflects an appropriate construct.

The construct validity of each subscale is also considered, not only in terms of the coherence of the factor structures, but also in terms of convergent and discriminant validity. The degree of convergent validation (variables correlating with variables they are expected to correlate with) and discriminant validity (where there are low correlations between different constructs) were also assessed [52], although the data were from the same method and multiple traits. That is, given the nature of the sample, multi-method, multi-trait analyses were unable to be performed and are an opportunity for future research.

A further contribution to the assessment of the validity of the derived instrument was in the form of assessments of predictive utility, a pragmatic assessment of whether the variables have practical worth and can predict outcomes of interest. The predictive utility of the subscales would be assessed through the use of multiple regression analyses on each of the outcome variables.

## 3. Results

### 3.1. Initial Structural Analyses

The overall starting list of items including their similarities between instruments are detailed in Appendix A. Exploratory factor analyses and confirmatory factor analyses were conducted. The exploratory factor analyses initially involved conducting principal components analyses to determine a number or range of the number of factors, then principal axis factor analyses using oblique (oblimin) rotation. The results from the exploratory factor analyses indicated that a large number of the items did not load as expected, with many items having low loadings (<|0.4|) on any factor, and the two factors expected to have the most items (per [4,5]) held only about half of their items. Further analyses were conducted using confirmatory factor analyses.

Structural equation modeling with maximum-likelihood estimation had more consistent results and provided clearer diagnostic information, especially regarding where cross-loading items should be positioned. That is, a variety of measurement model analyses were conducted, building up from one factor congeneric analyses to having multiple factors and then all of the resulting factors. The checks of the items on single factors and on sets of factors also ensured the unidimensionality of the factors and the discriminant validity of the factors (per [56]). The checks of unidimensionality also included assessments of misspecification for each item for each factor separately, assessments that each item and factor were discriminant from other factors (following [55]), and in terms of manual discrimination checks such as ensuring each item loaded on one factor more strongly than any item on other factors multiplied by the correlation between the factors.

The measurement model aimed to keep as many of the items as possible, although a few items did move from their initial factor to load more strongly and clearly on an alternate factor. The building up of the congeneric models to the overall measurement model led to three items being removed because they did not load clearly on any factor (items 5 and 11) or were very similar to, but not as clearly loading, as another item (item 4, which was effectively redundant to item 32).

Any item changes were not only assessed in terms of face validity, but also in terms of changes in the χ^2^ (i.e., Δχ^2^(df)) of the model (per [55]). Only changes that significantly improved the model and maintained face validity were applied. Changes that were indicated by high modification indices were only considered if they significantly improved the model, appeared to have face validity, and did not trigger any other concerns (such concerns as discussed in [55]). Where an item was moved to a different factor, further checks were also conducted as to whether the item could be moved elsewhere, but there were no instances where an item was moved more than once. Note that for all of the Δχ^2^(df) tests, the comparisons were only those that were fully nested.

Five items changed from their original factor to a new factor (items 42, 30, 21, 13, and 38, in order down Table 2 and Table A2). There were concerns that item 42 may not enhance the content validity of the recognition subscale, and instead, item 42 may load on recognition as a sequence effect where items 42 to 45 end up loading on the same factor. However, the loading of item 42 was checked in terms of potential misallocation and discriminant checks. Across all of the checks, 42 strongly remained on that factor, which seems to suggest that procedures that actively recognize the value of the nurses (such as by providing support (43), listening to their ideas (44), and providing floating staff (42)) may all be practical examples of such, further suggesting that item 42 may be in the right place. All of these items, except item 38, which was later removed entirely, remained on their new factors until the final measurement model.

Further analyses reviewed the measurement structure, excluding items with loadings less than 0.6 (shown in Table A2). That is, a further seven items (35, 36, 37, 50, 14, 38, and 48) were removed in order to tighten the loadings on the factors and enhance the construct validity of the factors, as well as to enhance the discriminant and convergent validity of the factors. The final, tighter set of factors used 40 of the initial 50 items, and these 40 items proceeded to form the scales assessed in the later analyses. The factor structure detailed in Table 2 below held up for a 60–40 hold-out sample analysis as well as for a combined sample analysis. There were no items with multiple loadings. There were no correlations between error terms allowed.

Further analyses found the structure was tighter without the two-item scales but would then have less coverage of the issues represented by those factors (peer work standards, orientation, positive scheduling climate), and therefore, less coverage of the construct domain of the nurse working environment. However, in those check analyses with all of the two-item factors removed, item 46 moves to nurse participation in hospital affairs. The factor structure on the right hand side of Table 2, retaining item 46, is used for the predictive utility checks (regressions) below, where the two-item scales are also used and often significant.

### 3.2. Scale-Level Reliability Analyses, and Convergent and Discriminant Relationships

In terms of the assessment of the reliabilities and then the convergent and discriminant validities of the scales, rather than the factors, Table 3 shows the Pearson correlation analyses that were conducted with the revised model and outcome variables. The reliability of the scales was assessed using Cronbach’s alpha coefficients. Most of the associations between these variables were statistically significant, revealing many positive and negative relationships among the variables analyzed. The strongest of these associations were five positive relationships between the variables of recognition and nurse participation in hospital affairs, nurse competence and nurse participation in hospital affairs, managers and nurse participation in hospital affairs, positive scheduling climate and recognition, and OCBO and IRB. More specifically, higher levels of recognition, nursing competence, and managers were related to higher levels of nurse participation in hospital affairs. Higher recognition was also associated with higher positive scheduling climate. Arguably, some of these relationships between facets of PEN may have been a little high, but the higher correlations indicate how the facets of PEN represented by the subscales are more similar to each other (suggesting convergent validity) than they are to the outcome measures (suggesting discriminant validity), yet all had varying relationships with the outcome variables in the later predictive utility checks.

Finally, as expected among outcome variables representing facets of the same domain of performance behavior, higher levels of OCBO were associated with higher levels of IRB. The remainder of the statistically significant relationships between the variables analyzed had moderate-to-weak associations. There were only a few associations that were not statistically significant, and these were mostly related to the OCBO and IRB variables (as would be expected, given that OCB and IRB are indicators of performance behaviors rather than indicators of nurse retention, which is the historical focus of the PEN scales).

### 3.3. Predictive Utility as Assessed via Regression Analyses

The predictive utility of the new scales was then assessed via multiple regression analyses using all of the revised model variables for each of the outcome variables. Table 4 summarizes the results of the multiple regression analyses for the revised model and outcome variables. The revised model used in the analyses explained a significant amount of variance for all of the outcome variables. The processes for conducting the regressions followed the standard checks, such as for multicollinearity, following [54].

More specifically, the regression model was significant for intent to quit (R²adj = 0.218, F (10,458) = 13.78, *p* < 0.001), with recognition, managers, and positive scheduling climate significant in this model. High scores on these variables were associated with lower levels on the intent to quit outcome. For affective organizational commitment, the regression model was significant (R²adj = 0.243, F (10,458) =15.72, *p* < 0.001), with one significant main effect found for the managers variables, whereby high scores on managers were linked with high affective organizational commitment scores. The regression model was significant for affective occupational commitment (R²adj = 0.279, F (10,458) = 18.72, *p* < 0.001), with significant main effects of work/peer standards, nursing competence, and resources. Increased scores on these variables were associated with increased scores on affective occupational commitment. The regression was significant for job satisfaction (R²adj = 0.217, F (10,458) = 13.76, *p* < 0.001), with high scores on work/peer standards, nursing competence, nurse–physician collaboration, and positive scheduling climate significantly associated with higher satisfaction levels.

The regression for OCBI was significant (R²adj = 0.128, F (10,458) =7.71, *p* < 0.001). High scores on work/peer standards, nursing competence, resources, and nurse–physician collaboration significantly related with high OCBI. Similarly, the model for OCBO was significant, with high levels of (R²adj = 0.159, F (10,458) = 9.68, *p* < 0.001), with work/peer standards, nursing competence, resources, and nurse–physician collaboration significantly linked to high OCBO scores. Finally, the model was significant for IRB (R²adj = 0.118, F (10,458) =7.16, *p* < 0.001), with significant main effects of recognition, work/peer standards, nursing competence, resources, and nurse–physician collaboration. Low levels of recognition and resources were associated with high IRB scores, while high scores on work/peer standards, nursing competence, and nurse–physician collaboration were associated with high IRB scores. For comparison purposes, correlation and regression analyses were conducted for the models of perceived nursing work environment [5] and PES-NWI [17] and can be found in Appendix B. For all of the scales in the revised structure and the overall regressions, the revised structure had superior predictive utility to both of the source instruments.

## 4. Discussion

A variety of measurement model analyses of the PNWE and PES-NWI instruments led to a more parsimonious combined instrument with superior validity on several outcome scales, including intent to quit, organizational and occupational commitment, job satisfaction, and performance-oriented behaviors. The measurement model process included congeneric analyses, building to a full model using 40 out of 50 items across the two source instruments. The revised set of scales had good-to-reasonable levels of reliability, had an appropriate set of correlations with stronger associations with the scales that would expect to have stronger relationships, and weaker relationships with the scales where there would be expected to be weaker relationships. The revised set of scales had superior predictive utility than either of the source instruments.

Rather than falling into one of the camps using one instrument, we recommend informing analyses of the nurse working environment by using a combined set of scales, taking scales from each of the two instruments, namely: nurse participation in hospital affairs, recognition, nursing process, peer work standards, nursing competence, orientation, managers, resources, nurse–physician collaboration, and positive scheduling climate. Essentially, the new set of items and scales broadens the coverage of the PES-NWI by adding extra scales to the most stable core items of the PES-NWI, particularly in areas where there may have been gaps, such as those associated with the scheduling of shift work. Given the strength of the core PES-NWI items and scales in terms of having strong relationships with a range of nurse, patient, and organizational outcomes [21], it is likely that the new structure delineated above will also be strongly related to those outcomes, although future research will need to verify that.

The structure derived above may need further checks, adjustments, and improvements. For example, it is possible that item 42 loaded where it did due to an order effect, where the sequence of the item relative to other items may need to be mixed up in future. That is, item 42 is originally from nursing competence, but it is listed in the survey near the other items of recognition in Table 1 above.

The topics of the two-item scales (peer work standards, orientation, positive scheduling climate) appear strongly and separately in the measurement structure. However, future research may wish to build on the tight structure above to add more items for the smaller scales if they desire. A possible driver for wanting to expand the coverage of the smaller scales is that related issues such as shift work have received substantial attention in nursing research (e.g., [57]), suggesting that these topics are important and worthy of inclusion in future studies.

Perhaps the main limitation of this study is that the sample was younger than the typical profile of nurses in Australia, most probably due to the sample source (an online panel) and method (an online survey). Future research may wish to assess the structure on a broader nurse sample. Another possible limitation is the Australian context and its associated issues, such as being relatively unionized and still primarily female. However, with hospitals in Australia and elsewhere moving to adopt Magnet accreditation, and particularly with little translation involved with the items, the scales should perform well across English-speaking contexts in developed countries, especially as nurse work environment scales should be increasingly comparable and transferable across countries, where those with a similar language should be a straightforward first step.

Another constraint is that the regressions only assessed the predictive utility of the new scales on constructs from individual nurses, albeit constructs that have received a lot of attention as being important predictors of Magnet hospitals, such as job satisfaction. Future research could extend that set of predictive checks to organizational performance, quality of care, and nurse turnover behavior, among other outcomes.

## 5. Conclusions

By essentially combining the best items from each instrument, a more comprehensive representation of the nurse practice environment is obtained with improved predictive utility. By building on this tested structure, future studies and interventions can inform and change elements of the nurse working environment in order to improve the retention of nurses in a context where there is a global shortage. Future research can build on this revised set of items with a validated structure and predictive utility to inform management and interventions, as well as assess their utility in predicting unit-level outcomes.

## Figures and Tables

**Table 1:** Characteristics of the sample.

Variable	Score	*n*	%
Gender	Male	83	18.1
	Female	376	81.9
Age	<25 years	24	5.2
	25–34 years	129	28.1
	35–39 years	69	15.0
	40–44 years	66	14.4
	45–49 years	52	11.3
	50–54 years	69	15.0
	55+ years	50	10.9
Organizational tenure	<1 year	33	7.2
	1–4 years	179	39.0
	5–9 years	134	29.2
	10–14 years	52	11.3
	15–19 years	29	6.3
	20–24 years	19	4.1
	25+ years	13	2.8
Nurse registration status	Enrolled/Division 2	154	33.6
	RN—All endorsements	189	41.2
	RN—Clinical specialist	63	13.7
	RN—Unit manager	18	3.9
	RN—Research	15	3.3
	RN—Nurse practitioner	20	4.4
Type of employment	Full-time	223	48.6
	Part-time	191	41.6
	Casual	41	8.9
	Sessional	2	0.4
	Fee for service	2	0.4
Work schedule	Morning shifts	113	24.6
	Afternoon shifts	49	10.7
	Night shifts	37	8.1
	Rotating shifts	209	45.5
	Other	51	11.1

Note: RN = Registered nurse.

**Table 2:** The final resulting factor structure including the loadings (>0.6) for each factor.

Tighter Resulting Instrument	β
**Nurse Participation in Hospital Affairs**	
23. Staff nurses are involved in the internal governance of the hospital	0.765
6. Opportunity for staff nurses to participate in policy decisions	0.691
17. Opportunities for advancement	0.69
27. Staff nurses have opportunity to serve on hospital and nursing committees	0.665
22. An active quality-assurance program (B)	0.707
15. A chief nursing executive is equal in power and authority to other top-level hospital executives	0.602
32. Active in-service/continuing-education program for nurses (B *)	0.661
46. Clinical nurse specialists who provide patient care consultation (B) ^#^	0.605
28. Nursing administrators consult with staff on daily problems and procedures (F)	0.727
**Recognition**	
43. Nursing staff are supported in pursuing degrees in nursing (G)	0.77
44. Support for new and innovative ideas about patient care (G)	0.78
45. Contributions that nurses make to patient care are publicly acknowledged (G)	0.73
42. Floating, so that staffing is equalized among units (G)	0.676
**Nursing Process**	
29. Written, up-to-date nursing care plans for all patients (A)	0.617
31. Use of nursing diagnoses (A)	0.74
30. Patient care assignments that foster continuity of care, i.e., the same nurse cares for the patient from one day to the next ^†^ (H)	0.715
**Peer Work Standards**	
39. Working with experienced nurses who know the hospital (G)	0.722
19. Working with nurses who are clinically competent (B)	0.747
**Nursing Competence**	
40. Standardized policies, procedures, and ways of doing things	0.658
18. A clear philosophy of nursing that pervades the patient care environment (H)	0.688
26. Nursing care is based on a nursing, rather than a medical, model (H)	0.644
**Orientation**	
41. A good orientation program for newly employed nurses (G)	0.822
25. A preceptor program for newly hired RNs (B)	0.727
**Managers**	
33. Nurse managers consult with staff on daily programs and procedures	0.768
10. A nurse manager who is a good manager and leader (C)	0.735
20. A nurse manager who backs up the nursing staff in decision making (C)	0.775
7. Supervisors use mistakes as learning opportunities, not criticism	0.676
3. A supervisory staff that is supportive of the nurses	0.722
21. Administration that listens and responds to employee concerns	0.751
**Resources**	
12. Enough staff to get the work done	0.774
9. Enough RNs on staff to provide quality patient care	0.745
1. Adequate support services allow me to spend time with my patients	0.628
8. Enough time and opportunity to discuss patient care problems with nurse	0.718
34. A satisfactory salary (D)	0.617
13. Praise and recognition for a job well done (C)	0.76
**Nurse–Physician Collaboration**	
16. A lot of teamwork between nurse and doctors	0.831
2. Physician and nurses have good working relationships	0.67
24. Collaboration between nurse and physicians	0.877
**Positive Scheduling Climate**	
47. Staff nurses actively participate in developing their work schedules	0.77
49. Flexible or modified work schedules are available	0.69

β = factor loading. ^†^ When PES-NWI was analyzed alone, these items dropped off. (A) = part of the nursing process scale, (B) = part of the nursing competence scale, (B *) = part of the nursing competence scale but text somewhat different to PES-NWI comparison item, (C) = part of the nursing management scale, (D) = extra item part of staffing and resource adequacy, (E) = extra item that is part of nurse–physician collaboration, (F) from PES-NWI nurse participation in hospital affairs, (G) from nursing competence, (H) from nursing foundations for quality of care, ^#^ item 46 becomes unstable in check analyses, as discussed in text.

**Table 3:** Mean (standard deviation), reliability coefficients, and correlations of the revised model’s scales and the outcome variables.

	M (SD)	α	1	2	3	4	5	6	7	8	9	10	11	12	13	14	15	16
1. Nurse participation in hospital affairs	22.45 (5.38)	0.89	1															
2. Recognition	9.63 (2.76)	0.83	0.76 ^#^	1														
3. Nursing process	7.65 (2.04)	0.73	0.61 ^#^	0.57 ^#^	1													
4. Work/peer standards	5.70 (1.38)	0.70	0.53 ^#^	0.48 ^#^	0.46 ^#^	1												
5. Nursing competence	8.23 (1.84)	0.70	0.70 ^#^	0.60 ^#^	0.61 ^#^	0.59 ^#^	1											
6. Orientation	5.26 (1.57)	0.75	0.64 ^#^	0.60 ^#^	0.43 ^#^	0.49 ^#^	0.59 ^#^	1										
7. Managers	15.03 (4.14)	0.88	0.81 ^#^	0.69 ^#^	0.58 ^#^	0.53 ^#^	0.69 ^#^	0.56 ^#^	1									
8. Resources	8.15 (1.49)	0.86	0.68 ^#^	0.49 ^#^	0.45 ^#^	0.49 ^#^	0.55 ^#^	0.48 ^#^	0.58 ^#^	1								
9. Nurse–physician collaboration	8.15 (1.99)	0.84	0.62 ^#^	0.49 ^#^	0.45 ^#^	0.49 ^#^	0.55 ^#^	0.48 ^#^	0.58 ^#^	0.55 ^#^	1							
10. Positive scheduling climate	4.98 (1.49)	0.69	0.68 ^#^	0.71 ^#^	0.47 ^#^	0.43 ^#^	0.55 ^#^	0.49 ^#^	0.64 ^#^	0.59 *	0.49 ^#^	1						
11. Intent to quit	18.82 (7.06)	0.91	−0.38 ^#^	−0.41 ^#^	−0.36 ^#^	−0.28 ^#^	−0.37 ^#^	−0.25 ^#^	−0.43 ^#^	−0.39 ^#^	−0.30 ^#^	−0.39 ^#^	1					
12. Affective organizational commitment	24.89 (5.42)	0.81	0.43 ^#^	0.40 ^#^	0.32 ^#^	0.28 ^#^	0.41 ^#^	0.31 ^#^	0.48 ^#^	0.42 ^#^	0.35 ^#^	0.40 ^#^	−0.67 ^#^	1				
13. Affective occupational commitment	23.08 (5.44)	0.94	0.40 ^#^	0.35 ^#^	0.32 ^#^	0.47 ^#^	0.47 ^#^	0.32 ^#^	0.39 ^#^	0.30 ^#^	0.37 ^#^	0.35 ^#^	−0.36 ^#^	0.36 ^#^	1			
14. Job satisfaction	21.38 (5.21)	0.91	0.37 ^#^	0.35 ^#^	0.31 ^#^	0.39 ^#^	0.41 ^#^	0.29 ^#^	0.38 ^#^	−0.31 ^#^	0.37 ^#^	0.37 ^#^	−0.59 ^#^	0.52 ^#^	0.62 ^#^	1		
15. OCBI	38.98 (6.31)	0.90	0.22 ^#^	0.15 ^#^	0.19 ^#^	0.31 ^#^	0.30 ^#^	0.19 ^#^	0.19 ^#^	0.25 ^#^	0.14 *	0.14 *	−0.13 ^#^	0.15 *	0.31 ^#^	0.32 ^#^	1	
16. OCBO	38.90 (6.17)	0.76	0.05	−0.01	0.09 *	0.24 ^#^	0.24 ^#^	0.08	0.06	0.19 ^#^	0.021	0.02	−0.20 ^#^	0.14 *	0.26 ^#^	0.27 ^#^	0.63 ^#^	1
17. In-role behaviors	40.94 (6.05)	0.77	0.11 *	0.00	0.11 *	0.23 ^#^	0.23 ^#^	0.11 *	0.07	0.18 ^#^	0.03	0.03	−0.12 *	0.09	0.21 ^#^	0.21 ^#^	0.63 ^#^	0.74 ^#^

* *p* < 0.05, ^#^ *p* < 0.001.

**Table 4:** Summary of regression analyses using the revised model scales as predictors.

Variables	Intent to Quit		Organizational Commitment		Occupational Commitment		Job Satisfaction		OCBI		OCBO		IRB	
	B (SE)	β	B (SE)	β	B (SE)	β	B (SE)	β	B (SE)	β	B (SE)	β	B (SE)	β
Nurse participation in hospital affairs	0.15 (0.12)	0.12 *	−0.03 (0.09)	−0.02	0.8 (0.09)	0.08	−0.03 (0.09)	−0.03	0.15 (0.11)	0.13	−0.10 (0.11)	−0.09	0.15 (0.11)	0.13
Recognition	−0.38 (0.19)	−0.15	0.11 (0.14)	0.06	0.01 (0.14)	0.00	0.09 (0.14)	0.05	−0.18 (0.18)	−0.08	−0.33 (0.17)	−0.15	−0.45 (0.17)	−0.21 *
Nursing process	−0.38 (0.20)	−0.11	−0.03 (0.15)	−0.01	−0.08 (0.15)	−0.03	0.02 (0.14)	0.01	0.05 (0.19)	0.02	−0.01 (0.18)	−0.00	0.01 (0.18)	0.00
Work/peer standards	−0.04 (0.28)	−0.01	−0.16 (0.21)	−0.04	10.06 (0.21)	0.27 ^#^	0.65 (0.21)	0.17 *	10.02 (0.26)	0.22 ^#^	0.95 (0.25)	0.21 ^#^	0.85 (0.25)	0.20 ^#^
Nursing competence	−0.25 (0.26)	−0.06	0.38 (0.20)	0.13	0.75 (0.19)	0.25 ^#^	0.44 (0.19)	0.16 *	0.71 (20.4)	0.21 *	10.15 (0.23)	0.34 ^#^	0.94 (0.24)	0.29 ^#^
Orientation	0.33 (0.26)	0.07	−0.09 (0.20)	−0.03	−0.10 (0.19)	−0.03	−0.12 (0.19)	−0.04	−0.01 (0.25)	−0.00	−0.07 (0.24)	−0.02	−0.01 (0.24)	−0.00
Managers	−0.33 (0.14)	−0.19 *	0.33 (0.10)	0.25 ^#^	0.04 (0.10)	0.03	0.09 (0.10)	0.07	−0.08 (0.127)	−0.05	−0.01 (0.12)	−0.01	−0.19 (0.12)	−0.13
Resources	−0.16 (0.11)	−0.09	0.12 (0.09)	0.09	−0.18 (0.08)	−0.14 *	−0.08 (0.08)	−0.07	−0.30 (0.11)	−0.19 *	−0.43 (0.10)	−0.28 ^#^	−0.25 (0.10)	−0.17 *
Nurse–physician collaboration	−0.07 (0.20)	−0.02	0.16 (0.15)	0.06	0.27 (0.15)	0.10	0.36 (0.15)	0.14 *	0.38 (0.19)	0.12 *	0.59 (0.18)	0.19 ^#^	0.41 (0.18)	0.14 *
Positive scheduling climate	−0.59 (0.30)	−0.12 *	0.38 (0.23)	0.10)	0.30 (0.22)	0.08	0.47 (0.22)	0.14 *	−0.05 (0.28)	−0.01	−0.02 (0.27)	−0.01	−0.16 (0.27)	−0.04
Adjusted R²	0.218		0.243		0.279		0.217		0.128		0.159		0.118	

* *p* < 0.05, ^#^ *p* < 0.001.

## Data Availability

The data are not publicly available due to privacy reasons.

## Appendix A

Appendix A includes a detailed summary of item matching and the non-overlapping items. Using the PES-NWI instrument [17] as a starting point, the items of PNWE [5] are compared in Table A1.

**Table A1:** The similar PNWE items matched to the PES-NWI items.

PES-NWI Instrument	PNWE Instrument
**Nurse Participation in Hospital Affairs**	**Professional Practice**
23. Staff nurses are involved in the internal governance of the hospital (e.g., practice and policy committees)	23. Staff nurses are involved in the internal governance of the hospital
6. Opportunity for staff nurses to participate in policy decisions	6. Opportunity for staff nurse to participate in policy decisions
17. Opportunities for advancement	17. Opportunities for advancement
21. Administration that listens and responds to employee concerns	21. Administration that listens and responds to employee concerns
11. A chief nursing officer who is highly visible and accessible to staff	
5. Career development/clinical ladder opportunity ^†^	5. Career development/clinical ladder opportunity
28. Nursing administrators consult with staff on daily problems and procedures	
27. Staff nurses have the opportunity to serve on hospital and nursing committees	27. Staff nurses have opportunity to serve on hospital and nursing committees
15. A chief nurse officer equal in power and authority to other top level hospital executives	15. A chief nursing executive is equal in power and authority to other top-level hospital executives
**Nursing Foundations for Quality of Care**	
31. Use of nursing diagnoses	31. Use of nursing diagnoses (A)
22. An active quality assurance program	22. An active quality-assurance program (B)
25. A preceptor program for newly hired RNs ^†^	25. A preceptor program for newly hired RNs (B)
26. Nursing care is based on a nursing, rather than a medical, model	
30. Patient care assignments that foster continuity of care, i.e., the same nurse cares for the patient from one day to the next ^†^	
18. A clear philosophy of nursing that pervades the patient care environment	
29. Written, up-to-date nursing care plans for all patients ^†^	29. Written, up-to-date nursing care plans for all patients (A)
14. High standards of nursing care are expected by the administration ^†^	
4. Active staff development or continuing-education programs for nurses	32. Active in-service/continuing-education program for nurses (B *)
19. Working with nurses who are clinically competent ^†^	19. Working with nurses who are clinically competent (B)
**Nurse Manager Ability, Leadership, and Support of Nurses**	
10. A nurse manager who is a good manager and leader	10. A nurse manager who is a good manager and leader (C)
20. A nurse manager who backs up the nursing staff in decision making, even if the conflict is with a physician	20. A nurse manager who backs up the nursing staff in decision making (C)
7. Supervisors use mistakes as learning opportunities, not criticism	
3. A supervisory staff that is supportive of the nurses	3. A supervisory staff that is supportive of nurses (C)
13. Praise and recognition for a job well done	13. Praise and recognition for a job well done (C)
**Staffing and Resource Adequacy**	**Staffing and Resource Adequacy**
12. Enough staff to get the work done	12. Enough staff to get the work done
9. Enough registered nurses to provide quality patient care	9. Enough RNs on staff to provide quality patient care
1. Adequate support services allow me to spend time with my patients	1. Adequate support services allow me to spend time with my patients
8. Enough time and opportunity to discuss patient care problems with other nurses	8. Enough time and opportunity to discuss patient care problems with nurse
**Collegial Nurse–Physician Relations**	**Nurse–Physician Collaboration**
16. A lot of teamwork between nurses and physicians	16. A lot of teamwork between nurse and doctors
2. Physicians and nurses have good working relationships	2. Physician and nurses have good working relationships
24. Collaboration (joint practice) between nurses and physicians	24. Collaboration between nurse and physicians
	**Nursing Management**
	33. Nurse managers consult with staff on daily program and procedures
	34. A satisfactory salary (D)
	**Nursing Process**
	35. Use of problem-oriented medical record
	36. Each nursing unit determines its own policies and procedures
	37. Team nursing as the nursing delivery system
	38. Physicians provide high-quality medical care (E)
	**Nursing Competence**
	39. Working with experienced nurses who know the hospital
	40. Standardized policies, procedures, and ways of doing things
	41. A good orientation program for newly employed nurses
	42. Floating, so that staffing is equalized among units
	43. Nursing staff are supported in pursuing degrees in nursing
	44. Support for new and innovative ideas about patient care
	45. Contributions that nurses make to patient care are publicly acknowledged
	46. Clinical nurse specialists who provide patient care consultation
	**Positive Scheduling Climate**
	47. Staff nurses actively participate in developing their work schedules
	48. Regular, permanently assigned staff nurses never have to float
	49. Flexible or modified work schedules are available
	50. Nursing care plans verbally transmitted from nurse to nurse

^†^ When PES-NWI analyzed these items dropped off, (A) = part of nursing process scale, (B) = part of nursing competence scale, (B *) = part of nursing competence scale but text somewhat different to PES-NWI comparison item, (C) = part of nursing management scale, (D) = extra item part of staffing and resource adequacy, (E) = extra item that is part of nurse–physician collaboration.

Each of the instruments were initially analyzed in terms of only their own pool of items. The PES-NWI factor structures were relatively consistent, with the overall structure holding as intended but with several items dropping off (items marked with † in the left-hand column in Table A1) because they did not load well on their factor and/or loaded similarly on multiple factors. The exception was item 22, which had a stronger loading on a different factor to that prescribed, moving from nursing foundations for quality of care to load on nurse participation in hospital affairs. Blank cells in the PNWE column represent where the PNWE did not have a similar item and vice versa.

**Table A2:** The overall factor structures and loadings per relevant factor prior to removal of low-loading items.

Broader Resulting Instrument	β
**Nurse Participation in Hospital Affairs**	
23. Staff nurses are involved in the internal governance of the hospital	0.766
6. Opportunity for staff nurses to participate in policy decisions	0.69
17. Opportunities for advancement	0.688
27. Staff nurses have opportunity to serve on hospital and nursing committees	0.667
22. An active quality-assurance program (B)	0.703
15. A chief nursing executive is equal in power and authority to other top-level hospital executives	0.603
32. Active in-service/continuing-education program for nurses (B *)	0.66
46. Clinical nurse specialists who provide patient care consultation (B)	0.607
28. Nursing administrators consult with staff on daily problems and procedures (F)	0.728
**Recognition**	
43. Nursing staff are supported in pursuing degrees in nursing (G)	0.769
44. Support for new and innovative ideas about patient care (G)	0.784
45. Contributions that nurses make to patient care are publicly acknowledged (G)	0.73
42. Floating, so that staffing is equalized among units (G)	0.672
**Nursing Process**	
29. Written, up-to-date nursing care plans for all patients (A)	0.58
31. Use of nursing diagnoses (A)	0.67
35. Use of problem-oriented medical record	0.595
36. Each nursing unit determines its own policies and procedures	0.529
37. Team nursing as the nursing delivery system	0.597
50. Nursing care plans verbally transmitted from nurse to nurse	0.571
30. Patient care assignments that foster continuity of care, i.e., the same nurse cares for the patient from one day to the next ^†^ (H)	0.636
**Peer Work Standards**	
39. Working with experienced nurses who know the hospital (G)	0.722
19. Working with nurses who are clinically competent (B)	0.723
14. High standards of nursing care are expected by the administration ^†^ (H)	0.516
38. Physicians provide high-quality medical care (E)	0.638
**Nursing Competence**	
40. Standardized policies, procedures, and ways of doing things	0.668
18. A clear philosophy of nursing that pervades the patient care environment (H)	0.683
26. Nursing care is based on a nursing, rather than a medical, model (H)	0.638
**Orientation**	
41. A good orientation program for newly employed nurses (G)	0.821
25. A preceptor program for newly hired RNs (B)	0.728
**Managers**	
33. Nurse managers consult with staff on daily program and procedures	0.766
10. A nurse manager who is a good manager and leader (C)	0.735
20. A nurse manager who backs up the nursing staff in decision making (C)	0.774
7. Supervisors use mistakes as learning opportunities, not criticism	0.677
3. A supervisory staff that is supportive of the nurses	0.723
21. Administration that listens and responds to employee concerns	0.752
**Resources**	
12. Enough staff to get the work done	0.775
9. Enough RNs on staff to provide quality patient care	0.744
1. Adequate support services allow me to spend time with my patients	0.628
8. Enough time and opportunity to discuss patient care problems with nurse	0.717
34. A satisfactory salary (D)	0.616
13. Praise and recognition for a job well done (C)	0.761
**Nurse–Physician Collaboration**	
16. A lot of teamwork between nurse and doctors	0.837
2. Physician and nurses have good working relationships	0.677
24. Collaboration between nurse and physicians	0.869
**Positive Scheduling Climate**	
47. Staff nurses actively participate in developing their work schedules	0.765
48. Regular, permanently assigned staff nurses never have to float	0.536
49. Flexible or modified work schedules are available	0.697

β = factor loading, ^†^ When PES-NWI was analyzed alone, these items dropped off. (A) = part of nursing process scale, (B) = part of nursing competence scale, (B *) = part of nursing competence scale but text somewhat different to PES-NWI comparison item, (C) = part of nursing management scale, (D) = extra item part of staffing and resource Adequacy, (E) = extra item that is part of nurse–physician collaboration, (F) from PES-NWI nurse participation in hospital affairs, (G) from nursing competence, (H) from nursing foundations for quality of care.

## Appendix B

For the purposes of comparison, the tables in this appendix use the original scales of the two instruments. The variance explained in terms of adjusted R^2^ is higher for all of the new scales in Table 4 above and for all of the outcome variables.

**Table A3:** Summary of regression analyses using the perceived nursing work environment (Choi et al., 2004) scales [5].

Variables	Intent to Quit		Organizational Commitment		Occupational Commitment		Job Satisfaction		OCBI		OCBO		IRB	
	B (SE)	β	B (SE)	β	B (SE)	β	B (SE)	β	B (SE)	β	B (SE)	β	B (SE)	β
Professional practice	−0.11 (0.08)	−0.126	0.06 (0.06)	0.091	0.04 (0.06)	0.060	0.00 (0.06)	0.000	−0.01 (0.08)	−0.014	−0.17 (0.08)	−0.213 *	−0.08 (0.08)	−0.096 *
Nursing process	−0.06 (0.14)	−0.030	0.03 (0.11)	0.020	0.07 (0.11)	0.043	0.08 (0.10)	0.048	0.04 (0.13)	0.020	−0.14 (0.13)	−0.076	−0.11 (0.13)	−0.060
Nursing competence	0.05 (0.14)	0.023	−0.05 (0.10)	−0.031	0.36 (0.10)	0.237 ^#^	0.21 (0.10)	0.142 *	0.45 (0.13)	0.259 ^#^	0.52 (0.13)	0.306 ^#^	0.48 (0.13)	0.285 ^#^
Nursing management	−0.37 (0.15)	−0.188 *	0.46 (0.11)	0.303 ^#^	0.16 (0.11)	0.103	0.24 (0.11)	0.163 *	−0.01 (0.14)	−0.007	0.11 (0.14)	0.062	−0.03 (0.14)	−0.019
Staffing and resourceAdequacy	−0.18 (0.13)	−0.085	0.10 (0.09)	0.060	−0.21 (0.10)	−0.132 *	−0.11 (0.09)	−0.073	−0.26 (0.12)	−0.141 *	−0.41 (0.12)	−0.225 ^#^	−0.20 (0.12)	−0.112
Nurse–physician collaboration	0.04 (0.17)	0.015	0.08 (0.12)	0.036	0.34 (0.12)	0.155 *	0.34 (0.12)	0.160 *	0.45 (0.16)	0.177 *	0.74 (0.15)	0.297 ^#^	0.57 (0.15)	0.235 ^#^
Positive scheduling climate	−0.43 (0.21)	−0.127 *	0.21 (0.16)	0.081	0.22 (0.16)	0.083	0.23 (0.16)	0.090	−0.00 (0.20)	−0.001	−0.08 (0.20)	−0.026	−0.24 (0.20)	−0.082
Adjusted R²	0.197		0.237		0.231		0.196		0.090		0.104		0.069	

^*^*p* < 0.05, ^#^ *p* < 0.001.

The new scales have a higher R^2^ than either of the previous main instruments and for all of the outcomes investigated here. The pattern of the higher R^2^ results of the new scales relative to the two former instruments provides further support for the superior predictive utility of the new scales.

**Table A4:** Summary of regression analyses using the PES-NWI (Lake, 2002) [17] scales.

	Intent to Quit		Organizational Commitment		Occupational Commitment		Job Satisfaction		OCBI		OCBO		IRB	
Model	B (SE)	β	B (SE)	β	B (SE)	β	B (SE)	β	B (SE)	β	B (SE)	β	B (SE)	β
Nurse participation in hospital affairs	−0.10 (0.09)	−0.086	0.09 (0.07)	0.102	−0.03 (0.07)	−0.030	−0.04 (0.07)	−0.043	−0.08 (0.09)	−0.074	−0.35 (0.08)	−0.355 ^#^	−0.19 (0.08)	−0.194
Nurse foundations for quality of care	−0.22 (0.10)	−0.148 *	0.06 (0.08)	0.056	0.48 (0.07)	0.428 ^#^	0.28 (0.07)	0.265 ^#^	0.49 (0.09)	0.375 ^#^	0.54 (0.09)	0.429 ^#^	0.48 (0.09)	0.389 ^#^
Nurse manager ability, leadership, support of nurses	−0.35 (0.16)	−0.173 *	0.39 (0.12)	0.251 ^#^	0.11 (0.12)	0.072	0.27 (0.12)	0.180 *	−0.04 (0.15)	−0.021	0.11 (0.14)	0.063	−0.14 (0.15)	−0.079
Staffing and resource adequacy	−0.27 (0.15)	−0.108	0.19 (0.11)	0.102	−0.12 (0.11)	−0.062	−0.07 (0.11)	−0.039	0.24 (0.14)	−0.108	−0.43 (0.13)	−0.200 ^#^	−0.19 (0.13)	−0.092
Collegial nurse–physician relations	−0.00 (0.20)	0.000	0.16 (0.15)	0.057	0.33 (0.15)	0.121 *	0.39 (0.15)	0.149 *	0.40 (0.19)	0.127 *	0.63 (0.18)	0.203 ^#^	0.46 (0.18)	0.150 *
Adjusted R²	0.199		0.240		0.248		0.205		0.110		0.128		0.084	

****p* < 0.05, ^#^ *p* < 0.001.
